# Physcion reduces lipid accumulation and prevents the obesity in mice

**DOI:** 10.1186/s12986-019-0362-7

**Published:** 2019-05-15

**Authors:** Seon-Jeong Lee, Su-Jung Cho, Eun-Young Kwon, Myung-Sook Choi

**Affiliations:** 1 Department of Food Science and Nutrition, Kyungpook National University, Daegu, 41566 South Korea; 2 Center for Food and Nutritional Genomics Research, Kyungpook National University, Daegu, 41566 South Korea

**Keywords:** Obesity, Physcion, Heaptic steatosis, Adiposity

## Abstract

**Background:**

Obesity increases the risk of metabolic dysfunction such as dyslipidemia, hypertension, and fatty liver. Physcion (PY) is an anthraquinone that reportedly has anti-inflammatory and anti-bacterial properties. However, few studies have addressed the effect of PY on high-fat diet-induced obesity in mice. The purpose of this study was to investigate the effects of PY on obesity.

**Methods:**

Male C57BL/6 J mice were randomly divided into three groups and fed normal diet (ND, 5% fat, w/w), high-fat diet (HFD, 20% fat, 1% cholesterol, w/w), and HFD supplemented with 0.002% PY (w/w) for 16 weeks. Obesity-related biomarkers were analyzed including whole body and white adipose tissue (WAT) weight, in addition to lipid and inflammatory factors in the plasma, feces, liver and epididymal WAT. Significant differences among the groups were determined using Student’s *t*-test. Differences were considered statistically significant at *p* < 0.05.

**Results:**

Body and WAT weights were significantly decreased by the PY supplement relative to the HFD groups. Energy expenditure was enhanced by the PY supplement, which led to ameliorate plasma lipids, adipokines, cytokines, and fecal lipids. Fatty acid (FA) synthesis decreased in the liver, while FA oxidation increased. Finally, lipid synthesis markedly decreased whereas lipolysis and oxidation increased in WAT.

**Conclusions:**

The PY supplement suppressed lipid accumulation in WAT and the liver by regulating enzyme and gene levels. These results indicate that PY can improve diet-induced obesity and its complications such as dyslipidemia, hepatic steatosis, and inflammation.

## Background

Obesity is a global public health concern in adults and children. The GBD 2015 obesity collaborators reported that obesity has received major attention in many countries, the effects of this attention on trends and the disease burden of obesity remain uncertain [1]. In recent years, rapid economic growth, increased consumption of fast food, and decreased physical activity have increased the number of individuals with high body weight (BW) and the incidence of obesity [2]. According to the World Health Organization (WHO), there were approximately 1.9 billion overweight adults, 650 million obese adults, and 41 million overweight and obese children in 2016. Obesity is a chronic disease and induces metabolic syndrome, such as cardiovascular disease, hypertension, type 2 diabetes, dyslipidemia and non-alcoholic fatty liver disease (NAFLD) [3]. One hallmark of obesity is the excessive accumulation of white adipose tissue (WAT). An imbalance of WAT is associated with many metabolic diseases and other clinical consequences that involve fatty acid (FA) fluxes, abnormal adipokine secretion, oxidative stress, and inflammatory responses.

Anthraquinones are a type of polyphenolic compound that is usually derived from Rumex and Rheum genus plants that are used as traditional medicines [4]. The main anthraquinones of the plants are chrysophanol, physcion (PY), emodin, and rhein [5]. PY was previously reported to have anti-inflammatory, anti-bacterial, and anti-fungal properties [6]. It was reported to inhibit protein tyrosine phosphatase 1B (PTP1B) that strongly enhanced insulin sensitivity by increasing both tyrosine phosphorylation of the insulin receptor and Akt phosphorylation [7].

The use of pharmacological agents to treat obesity is limited by their lack of efficacy and serious side effects. Alternative approaches that are safe and lower the risks associated with obesity are urgently required [8]. Recently, efforts are being made to find new bioactive compounds from natural resources which are abundant in environment and have few side effects. Therefore, the purpose of this study was to investigate effect of PY, as dietary supplement, on obesity and its related metabolic disorders in diet-induced obesity C57BL/6 J mice.

## Methods

### Animals and diets

The C57BL/6 J mice (4-week-old, male, *n* = 34) were purchased from Jackson Laboratory (Bar Harbor, USA). All mice were individually housed under a constant temperature (22 ± 2 °C) and 12-h light/dark cycle and fed a normal chow diet for 1 week after arrival for acclimation. At 5 weeks of age, mice were randomly divided into three groups and fed a normal diet (ND, *n* = 10, 5% fat, w/w), high-fat diet (HFD, *n* = 13, 20% fat and 1% cholesterol, w/w), or HFD with 0.002% PY (*n* = 11, Chemfaces, Wuhan, China, w/w) for 16 weeks. The mice had free access to food and distilled water during the experimental period. The composition of experimental diets in each group is shown in Table 1. Food intake and BW were measured once a week. For calculation of accurate food intakes, the same amount of diets was provided daily and amounts of leftover diets were checked to measure daily food consumption.

**Table 1:** Diet composition for animal experiments

Ingredient (g)	ND	HFD	PY
Casein	200.00	200.00	200.00
D,L-methionine	3.00	3.00	3.00
Corn starch	150.00	111.00	111.00
Sucrose	499.99	369.96	369.94
Cellulose power	50.00	50.00	50.00
Corn oil	50.00	30.00	30.00
Lard	–	170.00	170.00
Mineral mixture^1)^	35.00	42.00	42.00
Vitamin mixture^2)^	10.00	12.00	12.00
Choline bitartrate	2.00	2.00	2.00
Cholesterol	–	10.00	10.00
tert-Butylhydroquinone	0.01	0.04	0.04
Bioactive compound	–	–	0.02
Total (g)	1000	1000	1000

^1)^AIN-76 mineral mixture contained in g/kg of mixture: calcium phosphate dibasic, 500.00; sodium chloride, 74.00; potassium citrate H_2_O, 222.00; potassium sulfate, 52.00; magnesium oxide, 24.00; manganese carbonate, 3.50; ferric citrate U.S.P, 6.00; zinc carbonate, 1.60; cupric carbonate, 0.30; potassium iodate, 0.01; sodium selenite, 0.01; chromium potassium sulfate 12 H_2_O, 0.55; sucrose, finely powdered, 118.03

^2)^AIN-76 vitamin mixture contained in (g/kg of mixture): thiamine HCl 0.60; riboflavin, 0.60; pyridoxine HCl, 0.70; niacin, 3.00; calcium pantothenate, 1.60; folic acid, 0.20; biotin, 0.02, vitamin B12 (0.1%), 1.00; vitamin A palmitate (500,000 IU/g), 0.80; vitamin D3 (400,000 IU/g), 0.25; vitamin E acetate (500 IU/g), 10.00; menadione sodium bisulfate, 0.08; sucrose, finely powdered, 981.15. ND, normal diet (AIN-76, 5% fat, w/w); HFD, high-fat diet (20% fat, 1% cholesterol, w/w); PY, HFD + 0.002% physcion (w/w)

### Sampling

Mice were sacrificed using isoflurane (5 mg/kg BW, Baxter, USA) after a 12-h fast. Blood was collected in heparinized tubes from the vena cava, centrifuged at 1000×*g* for 15 min at 4 °C, and stored at − 70 °C before plasma profile analysis. The liver and adipose tissues were removed, rinsed in cold saline, patted dry, weighed, and stored at − 70 °C. Feces were collected during the 6 days, and dried feces were used for the determination of fecal lipid levels.

### Energy expenditure (EE) measurement

EE was measured using an indirect calorimeter (Oxylet; Panlab, Cornella, Spain). The mice were placed into individual metabolic chambers at 25 °C, with free access to food and water. O_2_ and CO_2_ analyzers were calibrated with highly purified gas. Oxygen consumption and carbon dioxide production were recorded at 3-min intervals using a computer-assisted data acquisition program (Chart 5.2; AD Instrument, Sydney, Australia) over 24 h, and the data were averaged for each mouse. EE was calculated according to the following formula;

EE (kcal/day/kg of body weight ^0.75^) = VO_2_ × 1.44 × [3.815 + (1.232 × VO_2_/VCO_2_)].

### Plasma, hepatic and fecal lipids profile analysis

The concentrations of plasma lipids were determined with the commercial kits. Triglyceride (TG), total cholesterol (Total-C), and high-density lipoprotein-cholesterol (HDL-C): Asan, Seoul, Republic of Korea; Free FA (FFA): Wako Pure chemical, Osaka, Japan; Apolipoprotein (apo) B: Nittobo medical co., LTD, Japan. The values of nonHDL-cholesterol (nonHDL-C) was calculated as follow: nonHDL-C = (Total-C)-(HDL-C).

Hepatic and fecal lipids were extracted [9], and dried lipids residues were dissolved in 1 mL of ethanol. Triton X-100 and a sodium cholate solution in distilled water were added to 200 μL of the dissolved lipid solution for emulsification. The TG, cholesterol and FA were analyzed with the same enzymatic kit used for the plasma lipids analysis.

### Plasma adipokine and cytokine analysis

Plasma adipokines (adiponectin, leptin) and cytokines (tumor necrosis factor (TNF)-α, monocyte chemotactic protein (MCP)-1, interleukin (IL)-6) were measured by MILLIPLEX®MAP Kit Mouse Adiponectin Magnetic Bead Single Plex and MILLIPLEX®MAP Kit Mouse Cytokine/Chemokine Magnetic Bead Panel (MERCK, New Jersey, USA), respectively.

### Lipid-regulating enzyme activities

The enzyme sources of liver and epididymal WAT were prepared according to the method reported by Hulcher F.H., and Oleson W.H. (1973) [10]. FA synthase (FASN) activity was determined by spectrophotometric assay according the methods of Nepokroeff C.M., Lakshmanan M.R., and Porter J.W. (1975) [11].; one unit of FASN activity represented the oxidation of 1 μmol of NADPH per minute at 37 °C. Phosphatide phosphohydrolase (PAP) activity was measured using the method of Walton P.A., and Possmayer F. (1985) [12] and PAP activity was expressed as mmol/mg protein/min. Malic enzyme (ME) activity was measured according to the method of Ochoa S. (1985) [13] by monitoring the production of NADPH at 340 nm, where the activity was represented by the formation of NADPH μmol/mg protein/min. FA β-oxidation was measured spectrophotometrically by monitoring the reduction of NAD to NADH in the presence of palmitoyl-CoA as described by Lazarow P.B. (1981) [14], with slight modification. Protein concentration was measured by the Bradford method using BSA as the standard [15].

### RNA extraction and real-time quantitative PCR analysis

Samples were prepared and analyzed as previously described [16]. Total RNA (1 μg) was reverse transcribed kit (Qiagen, Germany). Then mRNA expression was quantified by real-time quantitative PCR, using the QuantiTects SYBR green PCR kit (Qiagen, Germany) on the CFX96TM real-time PCR system (Bio-Rad, UK). Primers were used for detecting gene expression in liver and epididymal WAT, and the primer sequences were indicated in Table 2. The amplification was performed as follow: 10 min at 90 °C, 15 s at 95 °C and 60 s at 60 °C for a total of 35 cycles. The cycle threshold [17] values were normalized using GAPDH. Relative gene expression was calculated with the 2-Ct method.

**Table 2:** Primer sequences for genes used in real-time qPCR

Primer	Sequences
ACC1	5′-GGACAGACTGATCGCAGAGAA AG-3′ 5′-TGGAGAGCCCCACACACA-3′
ADRP	5′-GTGGAAAGGACCAAGTCTGTG-3′ 5′-GACTCCAGCCGTTCATAGTTG-3′
ADRB3	5′-ACCAACGTGTTCGTGACT-3′ 5′-ACAGCTAGGTAGCGGTCC-3’
CPT1	5′-ATCTGGATGGCTATGGTCAAGGTC-3′ 5′-GTGCTGTCATGCGTTGGCAGTC-3′
LIPE	5’-GGCTCACAGTTACCATCTCACC-3′ 5′-GAGTACCTTGCTGTCCTGTCC3’
PGC-1α	5′-AAGTGTGGA ACTCTCTGGAACTG-3′ 5′-GGGTTATCTTGGTTGGCTTTATG-3′
PGC-1β	5′-GGTCCCTGGCTGACATTCAC-3′ 5′-GGCACATCGAGGGCAGAG-3′
PPARα	5′-CCTGAACATCGAGTGTCGAATAT-3′ 5’-GGTCTTCTTCTGAATCTTGCAGCT-3’
PPARγ	5′-GAGTGTGACGACAAGATTTG-3′ 5′-GGTGGGCCAGAATGGCATCT-3′
PNPLA2	5’-CAACGCCACTCACATCTACGG-3′ 5’-TCACCAGGTTGAAGGAGGGAT-3′
SCD1	5′-CCCCTGCGGATCTTCCTTAT-3′ 5′-AGGGTCGGCGTGTGTTTCT-3′
SREBP-1a	5′-TAGTCCGAAGCCGGGTGGGCGCCGGCGCCAT-3′ 5′-GATGTCGTTCAA AACCGCTGTGTGTCCAGTTC-3′
SREBP-1c	5′- GGAGCCATGGATTGCACATT-3′ 5′-CCTGTCTCACCCCCAGCATA-3′
UCP1	5’-AGATCTTCTCAGCCGGAGTTT-3′ 5′-CTGTACAGTTTCGGCAATCCT-3’
GAPDH	5′-AAGGTCATCCCAGAGCTGAA-3′ 5′-CTGCTTCACCACCTTGA-3′

*ACC1* acetyl-CoA carboxylase 1, *ADRP* adipose differentiation-related protein, *ADRB3* beta-3 adrenergic receptor, *CPT1* carnitine palmitoyl-CoA transferase 1, *LIPE* lipase E, *PGC-1α and 1β* peroxisome proliferator-activated receptor gamma coactivator 1-alpha and beta, *PPARα* peroxisome proliferator activated receptor α, *PPARγ* peroxisome proliferator-activated receptor gamma, *PNPLA2* patatin-like phospholipase domain containing 2, *SCD1* stearoyl-CoA desaturase 1, *SREBP-1a and 1c* sterol regulatory element binding protein 1a and 1c, *UCP1* uncoupling protein 1, *GAPDH* glyceraldehyde-3-phosphate dehydrogenase

### Hepatic and adipose tissue morphology

The liver and epididymal WAT were removed from each mouse. Samples were fixed in 10% (v/v) paraformaldehyde/phosphate-buffered saline and embedded in paraffin for staining with hematoxylin and eosin. Stained areas were viewed using a microscope set at 200× magnification.

### Statistical analysis

Data were expressed as the mean ± standard error of the mean (SEM). All statistical analyses were performed using SPSS (SPSS, Inc., Chicago, IL, USA) for Windows. Significant differences among the groups were determined using student’s *t*-test. Differences were considered statistically significant at *p* < 0.05.

## Results

### BW and organ weight

**Fig. 1:**
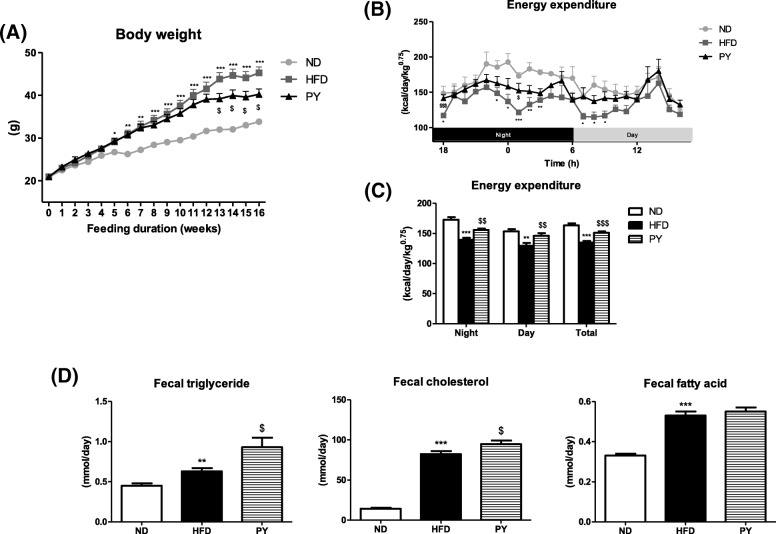
Change in body weight (**a**), energy expenditure (**b**&**c**), fecal lipid excretion (**d**). Data are mean ± S.E. Significant differences between HFD versus ND are indicated; ^*^p < 0.05, ^**^p < 0.01, ^***^p < 0.001. Significant differences between HFD versus PY are indicated; ^$^*p* < 0.05, ^$$^*p* < 0.01, ^$$$^*p* < 0.001. ND, normal diet (AIN-76, 5% fat, w/w); HFD, high-fat diet (20% fat, 1% cholesterol, w/w); PY, HFD + 0.002% physcion (w/w)

BW was monitored weekly for 16 weeks (Fig. 1a). BW significantly decreased in the PY group compared to that in the HFD group between 13 and 16 weeks. The BW gain (BWG) and food efficiency ratio (FER) were lower in the PY group than in the HFD group (Table 3). Similar to the BW trends, liver weight significantly decreased in the PY group compared to that in the HFD group. Additionally, muscle weight significantly increased. WAT weight was expressed as weight per 100 g BW. In the HFD-fed mice, WAT depot weight (including epididymal, perirenal, retroperitoneum, mesenteric, visceral, subcutaneous and interscapular depots) increased compared to that in the ND-fed mice. In the PY-fed mice, the weights of mesenteric, subcutaneous, and visceral WAT decreased relative to the HFD-fed mice. Therefore, the weight of total WAT was significantly reduced in the PY-fed mice compared to that in the HFD-fed mice (Table 3).

**Table 3:** Effect of 16-week PY treatment on BW, FER, and organ and WAT weights

	ND	HFD	PY
Initial Body Weight (g)	20.86 ± 0.30	20.97 ± 0.41	20.89 ± 0.31
Final Body Weight (g)	33.83 ± 0.61	45.25 ± 1.42^***^	40.24 ± 1.27^$^
Body Weight Gain (g)	12.96 ± 0.67	24.08 ± 1.19^***^	19.35 ± 1.13^$^
Body Weight Gain (g/day)	0.10 ± 0.01	0.19 ± 0.01^***^	0.16 ± 0.01^$^
Food Intake (g/day)	3.82 ± 0.08	3.04 ± 0.09^***^	2.87 ± 0.05
Energy Intake (kcal/day)	14.77 ± 0.30	13.78 ± 0.39	13.02 ± 0.23
FER	0.007 ± 0.000	0.014 ± 0.001^***^	0.012 ± 0.001^$^
Organ weight (g/ 100 g body weight)			
Liver	3.51 ± 0.07	5.48 ± 0.27^***^	4.43 ± 0.35^$^
Muscle	0.94 ± 0.02	0.72 ± 0.02^***^	0.78 ± 0.02^$^
WAT weight (g/ 100 g body weight)			
Epididymal WAT	3.85 ± 0.18	6.02 ± 0.21^***^	5.69 ± 0.18
Perirenal WAT	0.49 ± 0.02	0.92 ± 0.03^***^	0.84 ± 0.06
Retroperitoneum WAT	0.99 ± 0.03	1.28 ± 0.05^***^	1.32 ± 0.04
Mesentric WAT	1.61 ± 0.06	2.59 ± 0.14^***^	2.01 ± 0.14^$^
Visceral WAT	6.94 ± 0.23	10.81 ± 0.24^***^	9.86 ± 0.35^$^
Subcutaneous WAT	2.15 ± 0.12	4.56 ± 0.19^***^	3.69 ± 0.23^$$^
Interscapular WAT	1.38 ± 0.12	1.94 ± 0.10^***^	1.81 ± 0.08
Total WAT	10.47 ± 0.40	17.31 ± 0.38^***^	15.35 ± 0.55^$$^

Data are mean ± S.E. Significant differences between HFD versus ND are indicated; ^***^p < 0.001. Significant difference between HFD versus PY are indicated; ^$^*p* < 0.05, ^$$^*p* < 0.01. ND, normal diet (AIN-76, 5% fat, w/w); HFD, high-fat diet (20% fat, 1% cholesterol, w/w); PY, HFD + 0.002% physcion (w/w); FER, food efficiency ratio = body weight gain per day/energy intake per day. WAT, white adipose tissue; BW, body weight

Visceral WAT = (epididymal + perirenal + retroperitoneum + mesenteric) WAT;

Total WAT = (visceral + subcutaneous + interscapular) WAT

### EE

EE was measured for 24 h and the result is shown in Fig. 1b&c. EE was significantly higher in the PY group than in the HFD group at 6 PM and 1 AM (Fig. 1b). EE notably decreased in the HFD group relative to the ND group during both night and day. In contrast, EE significantly increased in the PY group during night and day compared to that in the HFD group (Fig. 1c). Therefore, the average EE level during the 24 h of experimental period was markedly lower in the HFD group than in the ND group, and these levels were significantly higher in the PY group than in the HFD group.

### Plasma and fecal lipid profiles

Supplementation of PY altered the plasma lipid profiles in the mice fed HFD. The concentration of FFA, TG, Total-C, HDL-C, nonHDL-C, and Apo B significantly increased in the HFD group relative to the ND group. In the PY group, the concentrations of FFA, Total-C, nonHDL-C, and Apo B significantly decreased compared to the HFD group (Table 4).

**Table 4:** Effect of PY on plasma lipid profiles over 16 weeks

	ND	HFD	PY
FFA (mmol/L)	0.62 ± 0.03	0.73 ± 0.02^**^	0.68 ± 0.02^$^
TG (mmol/L)	0.77 ± 0.05	1.10 ± 0.05^***^	1.10 ± 0.04
Total-C (mmol/L)	3.63 ± 0.11	6.38 ± 0.28^***^	5.08 ± 0.24^$$^
HDL-C (mmol/L)	1.68 ± 0.10	3.04 ± 0.09^***^	2.63 ± 0.14^$^
nonHDL-C (mmol/L)	1.94 ± 0.12	3.34 ± 0.23^***^	2.44 ± 0.13^$$^
Apo B (mg/dL)	4.14 ± 0.37	6.07 ± 0.40^**^	4.74 ± 0.26^$^

Data are mean ± S.E. Significant differences between HFD versus ND are indicated; ^**^p < 0.01, ^***^p < 0.001. Significant differences between HFD and PY are indicated; ^$^*p* < 0.05, ^$$^*p* < 0.01. ND, normal diet (AIN-76, 5% fat, w/w); HFD, high-fat diet (20% fat, 1% cholesterol, w/w); PY, HFD + 0.002% physcion (w/w); FFA, free fatty acid; TG, triglyceride; Total-C, total-cholesterol; HDL-C, HDL-cholesterol; nonHDL-C, nonHDL-cholesterol = (Total-C)-(HDL-C); Apo B, apolipoprotein B

The HFD group showed markedly elevated fecal lipid levels including TG, cholesterol, and FA compared to the ND group. In the PY group, fecal TG and cholesterol levels increased compared to those in the HFD group, but there was no significant difference in fecal FA level (Fig. 1d).

### Plasma adipokine and cytokine levels

**Fig. 2:**
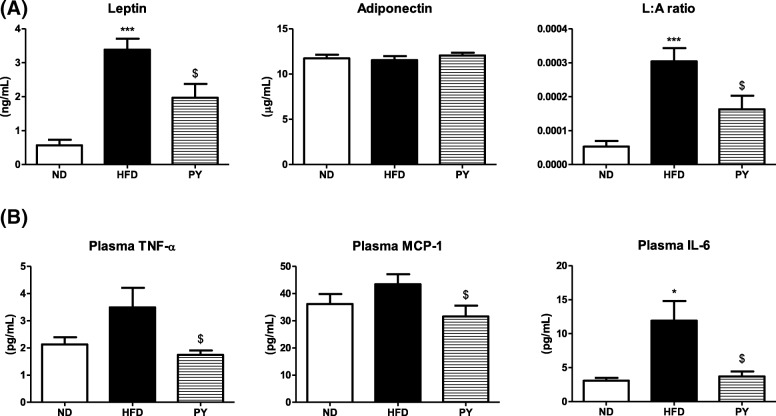
Effect of 16-week PY treatment on plasma adipokines (**a**), and plasma cytokines (**b**). Data are mean ± S.E. Significant differences between HFD and ND are indicated; ^*^p < 0.05, ^***^p < 0.001. Significant differences between HFD and PY are indicated; ^$^*p* < 0.05 ND, normal diet (AIN-76, 5% fat, w/w); HFD, high-fat diet (20% fat, 1% cholesterol, w/w); PY, HFD + 0.002% physcion (w/w); L:A, leptin:adiponectin; TNF-α, tumor necrosis factor-alpha; MCP-1, monocyte chemotactic protein-1; IL-6, interleukin-

As shown in Fig. 2a, there was no significant difference in adiponectin level between the three groups. Leptin and leptin:adiponectin (L:A) ratio levels significantly decreased in the PY group compared to those in the HFD group. Concentrations of MCP-1, TNF-α, and IL-6 were markedly lowered by PY supplementation (Fig. 2b).

### Hepatic lipid profiles, lipid-regulating enzyme activities, gene expression and morphology

**Fig. 3:**
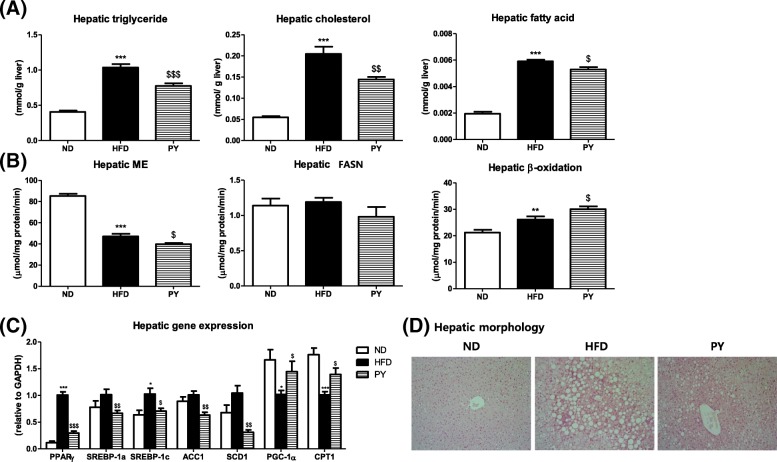
Effect of 16-week PY diet on hepatic lipid profiles. Hepatic lipid levels (**a**). Lipid-regulating enzyme activities (**b**). Lipid-metabolism related genes (**c**). Morphology (**d**). Data are mean ± S.E. Significant differences between HFD versus ND are indicated; ^*^p < 0.05, ^**^p < 0.01, ^***^p < 0.001. Significant differences between HFD versus PY are indicated; ^$^*p* < 0.05, ^$$^*p* < 0.01, ^$$$^*p* < 0.001. ND, normal diet (AIN-76, 5% fat, w/w); HFD, high-fat diet (20% fat, 1% cholesterol, w/w); PY, HFD + 0.002% physcion (w/w); ME, malic enzyme; FASN, fatty acid synthase; PPAR γ, peroxisome proliferator-activated receptor gamma; SREBP-1a and 1c, sterol regulatory element binding protein 1a and 1c; ACC1, acetyl-CoA carboxylase 1; SCD1, stearoyl-CoA desaturase 1; PGC-1α, peroxisome proliferator-activated receptor gamma coactivator 1-alpha; CPT1, carnitine palmitoyl-CoA transferase 1. Hematoxylin and Eosin (H&E) stained transverse-section of liver. Representative photomicrographs of liver are shown at 200x

Hepatic TG, cholesterol, and FA levels were higher in the HFD group than in the ND group. Similar to the trends observed in liver weight, PY supplementation dramatically decreased hepatic TG, cholesterol, and FA levels (Fig. 3a). Hepatic tissue morphology is shown in Fig. 3d. In the HFD group, there were large-sized hepatic lipid droplets compared to the ND group. However, the PY treatment reduced both hepatic lipid droplet number and size compared to HFD.

Figure 3b presents the activities of hepatic lipid-regulating enzyme. Although there were no statistical differences in FASN among the groups, ME activity significantly decreased in the PY group compared to that in the HFD group. β-oxidation activity increased in the PY supplementation group relative to the HFD group.

Figure 3c shows the expression levels of hepatic lipid metabolism-related genes. PY supplementation markedly reduced lipogenic gene expression levels including PPARγ, SREBP-1a, SREBP-1c, ACC1, and SCD1. CPT1 and PGC-1α are genes related to FA oxidation. The expression of CPT1 and PGC-1α was down-regulated in the HFD group compared to that in the ND group. However, PY up-regulated these genes compared to HFD.

### Lipid-regulating enzyme activities, gene expression and morphology in epididymal WAT

**Fig. 4:**
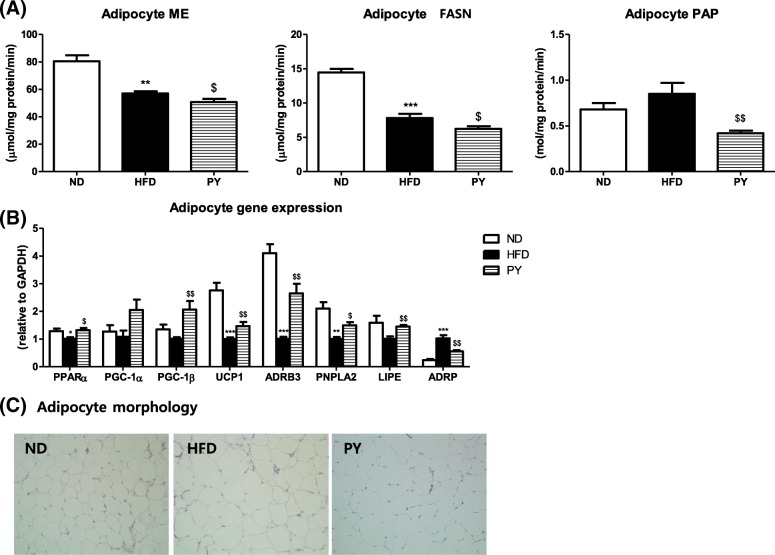
Effect of 16-week PY treatment on lipid profiles in epididymal WAT. Lipid-regulating enzyme activities (**a**). Lipid-metabolism related genes (**b**). Morphology (**c**). Data are mean ± S.E. Significant differences between HFD and ND are indicated; ^*^p < 0.05, ^**^p < 0.01, ^***^p < 0.001. Significant differences between HFD versus PY are indicated; ^$^*p* < 0.05, ^$$^*p* < 0.01 ND, normal diet (AIN-76, 5% fat, w/w); HFD, high-fat diet (20% fat, 1% cholesterol, w/w); PY, HFD + 0.002% physcion (w/w); ME, malic enzyme; FASN, fatty acid synthase; PAP, phosphatidate phosphohydrolase; PPARα, peroxisome proliferator activated receptor α; PGC-1α and 1β, peroxisome proliferator-activated receptor gamma coactivator 1-alpha and beta; UCP1, uncoupling protein 1; ADRB3, beta-3 adrenergic receptor; PNPLA2, patatin-like phospholipase domain containing 2; LIPE, lipase E; ADRP, adipose differentiation-related protein. Hematoxylin and Eosin (H&E) stained transverse-section of adipose tissue. Representative photomicrographs of liver are shown at 200x

Lipid-regulating enzyme activities and epididymal WAT morphology are indicated in Fig. 4a and c, respectively. PY significantly decreased the activities of ME, FASN, and PAP compared to HFD. Adipocyte size increased in HFD-fed mice compared to that in ND-fed mice, whereas PY supplementation reduced adipocyte size relative to HFD.

The expression of lipid regulating genes is indicated in Fig. 4b. Gene expression of PPARα, UCP1, and ADRB3, FA oxidation-related genes, decreased in the HFD group compared to that in the ND group. With PY supplementation, PPARα, PGC-1β, and UCP1 expression was significantly down-regulated and PGC-1α expression was tended to be increased. PNPLA2 and LIPE genes were associated with lipolysis. The expressions of PNPLA2 was significantly decreased in the HFD group compared to the ND group. The expression of PNPLA2 and LIPE were significantly increased in the PY group relative to the HFD group. ADRP, a gene involved in LD formation, was up-regulated by HFD compared to ND. In the PY group, the ADRP expression was markedly reduced compared to that in the HFD group.

## Discussion

Chronic HFD feeding induces obesity, which is a growing problem and is associated with metabolic complications [18, 19]. Anthraquinones are present in many medicinal and nutritional plants and PY is member of the anthraquinone family [20]. In this study, we elucidated the efficacy of PY on obesity via lipid metabolism in mice.

### Effect of PY supplementation on the regulation of lipid metabolism in the liver, plasma, and feces

Accumulation of TGs in the liver induces hepatic steatosis, which is a feature of NAFLD. Hepatic steatosis is also associated with an increased risk of dyslipidemia. HFD induces hepatic steatosis through multiple pathways, including increased dietary and released adipocyte FAs, excess hepatic FA synthesis, and reduced hepatic FA oxidation [21]. Hepatic de novo lipogenesis is regulated by the activity of lipogenic enzymes (ACC, FASN, SCD1), and transcription factor expression levels (SREBPs and PPARs) [22].

PPARγ is required for lipid synthesis, transport, and storage, and its expression is markedly increased in hepatic steatosis [23]. In our study, the expression levels of SREBP-1a, SREBP-1c, ACC1, SCD1, and PPARγ were significantly decreased by PY supplementation, ME activity was decreased compared to that in the HFD group, and hepatic lipid droplet size and number were reduced, presumably owing to its anti-lipogenic effects. Liver weight was significantly reduced in the PY group compared to that in the HFD group; similarly, hepatic TG, cholesterol, and FA levels decreased.

Liver mitochondria are the primary site for energy production in the liver through β-oxidation of FAs. Lipid oxidation-related genes were associated with the transcriptional control of PGC-1α [24].

Furthermore, regulation of β-oxidation depends on the delivery rate of FAs into the mitochondrial matrix, which is dependent on CPT1 activity [22]. The FA oxidation-related genes (PGC-1α, CPT1) were up-regulated by PY supplementation. Similar to results of gene expression, activity of β-oxidation was increased by the PY group. These results indicate that the PY supplementation ameliorates hepatic steatosis by increasing lipid oxidation resulting in a reduction of lipid accumulation.

Obesity is often associated with impaired lipolysis and increased plasma FFA concentrations. Abnormally high FFA levels stimulate the production of VLDL-TG in the liver; increased plasma lipids cause dyslipidemia [25]. Dyslipidemia in obesity is generally characterized by decreased HDL-C and increased LDL-C levels [26]. In the present study, plasma lipid profiles showed dramatic changes in the HFD group compared to ND, with PY supplementation leading to suppressed levels of FFA, Total-C, nonHDL-C, and Apo B compared to HFD. Our results were in agreement with a previous report on PY-containing herb extracts, which lowered cholesterol and LDL-C levels [7]. Additionally, PY supplementation increased the excretions of fecal TG compared to the HFD group. Therefore, the PY group decreased the absorption of dietary fat.

### Effect of PY supplementation on the regulation of fat mass, WAT lipid metabolism, and plasma adipokines

Increased fat mass is a main symptom of obesity, and is related to various obesity-related metabolic syndromes [27]. The several lipogenic enzymes, including FASN and ME, newly synthesize FAs that are used as substrates in TG synthesis [28]. In our results, FASN and ME activities were markedly suppressed in the PY group. Moreover, PAP activity, a rate-limiting enzyme in TG synthesis, was significantly attenuated by PY supplementation.

Increasing TG lipolysis and FA utilization might be useful approaches to improve or prevent obesity [29]. The stimulation of ADRB3 enhances lipolysis through lipid oxidation and thermogenesis [30]. In adipose tissue, TG hydrolysis requires lipases including HSL and ATGL (PNPLA2), and β-adrenergic stimulation activates HSL (LIPE). HSL translocates to lipid droplets and cooperates with ATGL to accelerate lipolysis. In general, beta-adrenergic receptor-stimulated lipolysis is impaired in obese patients and mice lacking ATGL have increased amounts of fat [31, 32]. Additionally, ATGL-deficient animals have decreased PPARα signaling [33]. PPARα is essential for activating lipid oxidation-related genes and interacts with PGC-1α to produce brite (brown in white) adipocytes. These pathways enhance UCP1 expression, which regulates EE and thermogenesis, and its expression is increased during FA oxidation [30, 34]. In our study, the FA oxidation-related genes (ADRB3, PGC-1β, UCP1) and lipolysis-related genes (PNPLA2, LIPE) were up-regulated by PY. ADRP, which stimulates lipid droplet formation, was down-regulated with a corresponding reduction in adipocyte size.

The PY supplement also increased EE, which is the amount of energy used, due to up-regulation of UCP1. Muscle weight also increased in the PY group. Meanwhile, visceral fat accumulation is highly linked with excess body fat, dyslipidemia, and hepatic steatosis. Our results show PY supplementation reduced visceral WAT, total WAT, and BW. It also reduced adipocyte size as observed via examination of WAT morphology compared to HFD. These results indicate PY supplementation enhances lipolysis, suppresses lipid accumulation, and ameliorates adiposity by regulating gene expression and EE.

Additionally, the excessive fat in obesity, especially visceral fat, produces cytokines including MCP-1, IL-6, and TNF-α, and adipokines such as leptin, which regulate pro-inflammatory cytokines [3, 35]. In this study, WAT reduction subsequently reduced plasma leptin levels, which we speculate could decrease plasma levels of MCP-1, IL-6, and TNF-α.

Taken together, the current evidence indicates that PY supplementation suppressed adiposity by reducing lipid droplet synthesis and increasing ADRB3-related lipolysis and EE via FA oxidation. In addition, it alleviates plasma levels of inflammatory cytokines and adipokines.

## Conclusions

The present study evaluated the effects of PY supplementation on diet-induced obesity and obesity-related metabolic disorders. Our data indicated that PY ameliorates hepatic steatosis by modulating lipid metabolism. Hepatic lipids and liver weight decreased with control of lipogenic-related gene (PPARγ, SREBP-1a, SREBP-1c, ACC1, SCD1) expression and enzyme activity (ME). In addition, PY enhanced FA oxidation-associated gene (PGC-1α, CPT1) expression and β-oxidation activity. Its effects on lipid metabolism in adipocytes included enhancing the expression of lipolysis-related genes (PNPLA2, LIPE) and thermogenesis-related genes (PPARα, PGC-1β, UCP1). Furthermore, night and day EE increased with higher muscle weight.

PY supplementation led to decreased lipogenic enzyme (ME, FASN, PAP) activity and ADRP expression, which suppressed lipid droplet formation. This culminated in reduced adipocyte size and body and fat weights. This treatment also reduced plasma lipids (FFA, Total-C, nonHDL-C, Apo B) and fecal lipids (TG, cholesterol) thereby improving the inflammatory response by reduction of plasma cytokines.

**Fig. 5:**
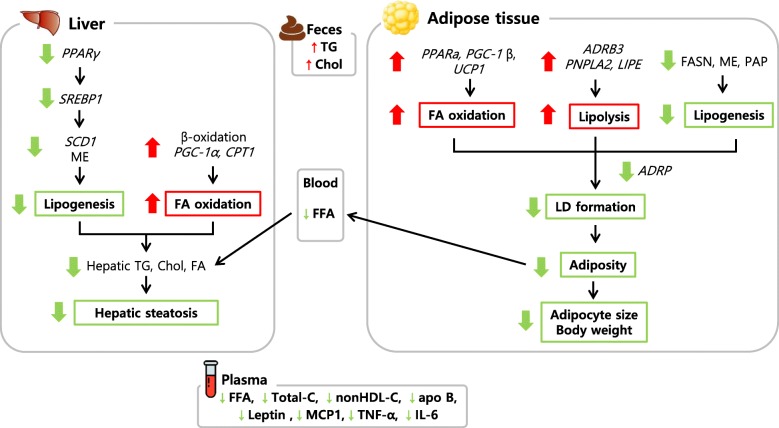
Our proposed mechanism of PY supplementation in liver and adipose tissue

Taken together, PY supplementation suppressed lipid accumulation and prevented a variety of obesity-related metabolic disorders including dyslipidemia, fatty liver, and inflammation. The proposed anti-obesity mechanisms of PY are shown in Fig. 5. Therefore, PY is a very potent bioactive compound that is a promising anti-obesity agent.

## Abbreviations

ACC1: Acetyl-CoA carboxylase 1
ADRB3: Beta-3 adrenergic receptor
ADRP: Adipose differentiation-related protein
Apo B: Apolipoprotein B
BW: Body weight
CPT1: Carnitine palmitoyl-CoA transferase 1
FASN: Fatty acid synthase
FER: Food efficiency ratio
FFA: Free fatty acid
GAPDH: Glyceraldehyde-3-phosphate dehydrogenase
H&E: Hematoxylin and eosin
HDL-C: high-density lipoprotein-cholesterol
IL-6: Interleukin-6
L:A: leptin:adiponectin
LIPE: Lipase E
MCP-1: Monocyte chemotactic protein-1
ME: Malic enzyme
nonHDL-C: nonHDL-cholesterol
PAP: Phosphatidate phosphohydrolase
PGC-1α: Peroxisome proliferator-activated receptor gamma coactivator 1-alpha
PGC-1β: Peroxisome proliferator-activated receptor gamma coactivator 1-beta
PNPLA2: Patatin-like phospholipase domain containing 2
PPAR γ: Peroxisome proliferator-activated receptor gamma
PPARα: Peroxisome proliferator activated receptor α
PTP1B: protein tyrosine phosphatase 1B
SCD1: Stearoyl-CoA desaturase 1
SEM: Standard error of the mean
SREBP-1a: Sterol regulatory element binding protein 1a
SREBP-1c: Sterol regulatory element binding protein 1c
TNF-α: Tumor necrosis factor-alpha
Total-C: Total cholesterol
UCP1: Uncoupling protein 1
